# deepPGSegNet: MRI-based pituitary gland segmentation using deep learning

**DOI:** 10.3389/fendo.2024.1338743

**Published:** 2024-02-02

**Authors:** Uk-Su Choi, Yul-Wan Sung, Seiji Ogawa

**Affiliations:** ^1^ Medical Device Development Center, Daegu-Gyeongbuk Medical Innovation Foundation, Daegu, Republic of Korea; ^2^ Kansei Fukushi Research Institute, Tohoku Fukushi University, Sendai, Japan

**Keywords:** MRI, pituitary gland, segmentation, deep learning, 3D UNet, pituitary disorders

## Abstract

**Introduction:**

In clinical research on pituitary disorders, pituitary gland (PG) segmentation plays a pivotal role, which impacts the diagnosis and treatment of conditions such as endocrine dysfunctions and visual impairments. Manual segmentation, which is the traditional method, is tedious and susceptible to inter-observer differences. Thus, this study introduces an automated solution, utilizing deep learning, for PG segmentation from magnetic resonance imaging (MRI).

**Methods:**

A total of 153 university students were enrolled, and their MRI images were used to build a training dataset and ground truth data through manual segmentation of the PGs. A model was trained employing data augmentation and a three-dimensional U-Net architecture with a five-fold cross-validation. A predefined field of view was applied to highlight the PG region to optimize memory usage. The model’s performance was tested on an independent dataset. The model’s performance was tested on an independent dataset for evaluating accuracy, precision, recall, and an F1 score.

**Results and discussion:**

The model achieved a training accuracy, precision, recall, and an F1 score of 92.7%, 0.87, 0.91, and 0.89, respectively. Moreover, the study explored the relationship between PG morphology and age using the model. The results indicated a significant association between PG volume and midsagittal area with age. These findings suggest that a precise volumetric PG analysis through an automated segmentation can greatly enhance diagnostic accuracy and surveillance of pituitary disorders.

## Introduction

One of the important endocrine organs located at the base of the brain is the pituitary gland (PG). It is responsible for the regulation of secretion of various hormones that control many vital functions of the body, such as growth, metabolism, reproduction, and stress response (1–3). The PG consists of two main lobes: the anterior lobe (adenohypophysis) and the posterior lobe (neurohypophysis), which possess different embryological origins and endocrine functions (1, 4).

Pituitary disorders are a heterogeneous group of conditions that affect the structure or function of the PG or its associated organs such as the hypothalamus (5). They can induce various clinical manifestations depending on the type and severity of hormone deficiency or excess, including a mass effect from pituitary tumors or lesions (6–9). These conditions can significantly impact physical health and quality of life including patients’ psychological well-being (10).

Magnetic resonance imaging (MRI) is the most widely used imaging modality to diagnose and monitor PG-related diseases such as tumor or mood disorders (11–13) as MRI can provide high-resolution images of the PG including its surrounding structures, such as the optic chiasm, cavernous sinuses, and carotid arteries (8, 14, 15). Through this modality, small changes in signal intensity or enhancement patterns that reflect different pathological processes of the PG can be detected (15–17).

Previous studies have reported variations in the pituitary gland (PG) volume measured via MRI among individuals with different health conditions (18), with it being smaller in patients with hypochondriasis or Prader–Willi syndrome compared to healthy individuals (19, 20) thus indicating distinct stress-induced responses of PG. The PG volume typically increases until early adulthood, followed by a decline, with some increase observed in women aged 50-59 (21). During puberty, especially in girls, the PG volume significantly increases (22). It can also undergo a drastic increase during pregnancy, returning to its normal size within 6 months (23, 24). Some evidence suggests an association between PG morphometry and function. Wu et al. reported a positive correlation between PG volume and hormones in the idiopathic central precocious puberty group (22), while Low et al. found that patients with isolated growth hormone deficiency have a smaller PG volume than controls due to abnormalities in the hormone system (25). Thus, structural characteristics of the PG, such as the volume, shape, location, and intensity features of the regions (26) using MRI became candidates of crucial biomarkers for evaluating the endocrine-related psychotic and physiologic function of the PG (27, 28).

Segmentation is a prerequisite for the evaluation of the structural characteristics of the PG, which render the quantitative analysis of the characteristics possible. Segmentation can also allow integration of structural and functional information by enabling signal extraction from specific regions of interest (ROIs) in functional MRI (fMRI) analysis (26). Thus, segmentation is vital for valuable insights into the normal and abnormal physiology of the PG and its related networks (26, 29).

Majority of the previous studies used manual segmentation; however, it is time-consuming, tedious, and prone to human errors (30–32), and a semi-automatic method using MRI data resampling, image filtering, and region-growing was adopted to segment the PG efficiently (33, 34). However, even a semi-automatic segmentation is a daunting task due to several factors (26): 1) The small size and complex shape of the PG; 2) the anatomical and pathological variations among individuals; 3) the low contrast between different tissues; and 4) the lack of standardized criteria or protocols for segmentation.

Recently, deep-learning-based methods for segmentation of the PG, which could cope with those challenging factors, have been developed (9, 35, 36). Deep learning is a branch of machine learning that uses artificial neural networks with multiple layers to learn complex patterns or features from a large amount of data (37, 38). Deep learning has demonstrated remarkable performance in various image processing tasks such as classification, detection, and especially segmentation (38). These deep-learning-based segmentation methods have exhibited robust performances for an abnormal PG such as pituitary adenomas with varying architectures (9, 35, 36). However, those methods are not applicable to normal PG segmentation although the morphometry of the normal PG is equally important for the evaluation of health conditions such as obesity, growth disorders, and abnormal morphometry such as adenomas (2, 5, 17, 25). A recent report indicated that obese patients exhibited a larger PG volume compared to both normal and overweight subjects (5). This suggests that the morphometry of the PG could serve as a biomarker for predicting dysfunction in the pituitary system. The segmentation of the normal PG morphometry can provide a distribution within a specific control group, serving as a baseline for calculating abnormalities.

In this study, we aimed to develop and evaluate a deep-learning-based method for automated segmentation of the normal PG from MRI scans, which is referred to as deepPGSegNet. We trained a dataset of normal university student MRI images employing a three-dimensional (3D) UNet architecture with a limited ROI strategy. The proposed model was robustly evaluated using dice coefficient (DICE) loss, Interaction of Union (IoU), and DICE during five-fold cross-validation. Additionally, we estimate the association between PG morphologies and chronological ages using an external dataset of MRI images of normal participants.

## Methods

### Participants for model construction and evaluation

A total of 153 right-handed university students (55 male, 98 females; age range: 20–23 years) participated in this study. They had no neurological or medical problems and provided informed consent. We used 143 out of 153 participants as participants for the creation and evaluation of the PG segmentation model. The ten participants randomly selected from a pool of 153 were used to test the segmentation model.

### Participants for regression of PG length with chronological age

A total of 29 participants (11 males, 18 females; age range: 21–68 years) were included in this study, with four or more participants for each age group when the age interval was set at 10 years. They had no neurological or medical problems and provided informed consent. The study was approved by the Institutional Review Board of Tohoku Fukushi University. We used 29 participants for the evaluation of PG morphometry on chronological ages.

### MRI parameters

All 182 participants were scanned using a 3T Skyra-fit MRI scanner with a 20-channel head coil. We acquired T1-weighted (T1w) images through a magnetization prepared rapid acquisition with gradient echo sequence (repetition time = 1900 ms, echo time = 2.52 ms, flip angle = 80°, number of slices = 192, slice thickness = 1 mm, matrix = 256 × 256, and in-plane voxel resolution = 1 × 1 mm^2^).

### Training data preparation

**Figure 1:**
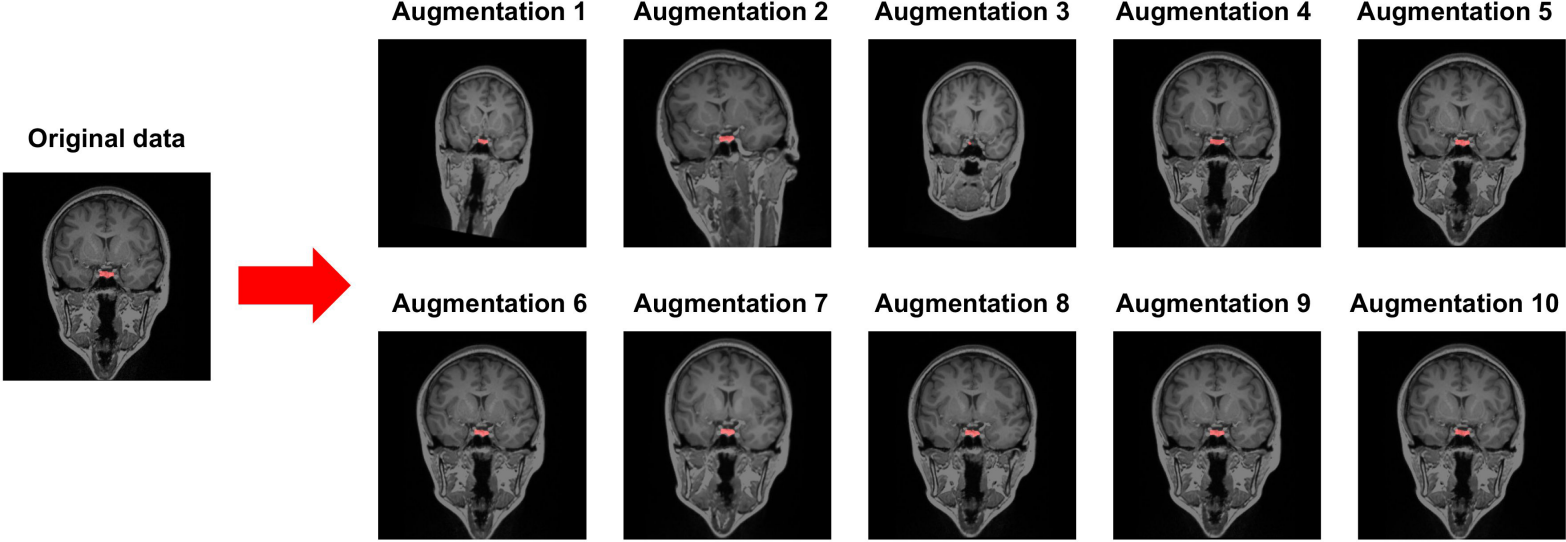
Data augmentation using random affine transformation and random elastic deformation. Red cluster indicates a manually segmented pituitary gland.

A dataset of MRI brain images from 153 participants using ITK-SNAP software (http://www.itksnap.org) that has been widely used in medical imaging analysis was manually segmented. The dataset was divided into two sets: a training set of 143 participants and a test set of 10 participants. An augmentation using random affine and random elastic deformation methods was employed to the training dataset to expand the sample size of the training data (**Figure 1**). To determine the location of the center voxel for the training ROI, we calculated the average x, y, and z-axis coordinates of the manually segmented PG images.

### Model training

**Figure 2:**
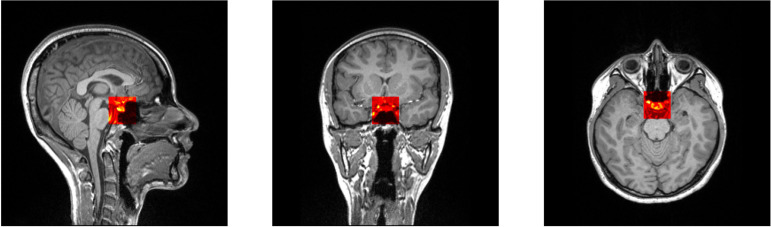
Selected patch with 32 × 32 × 32 size from predefined coordinates (x = 128, y = 137, z = 131).

**Figure 3:**
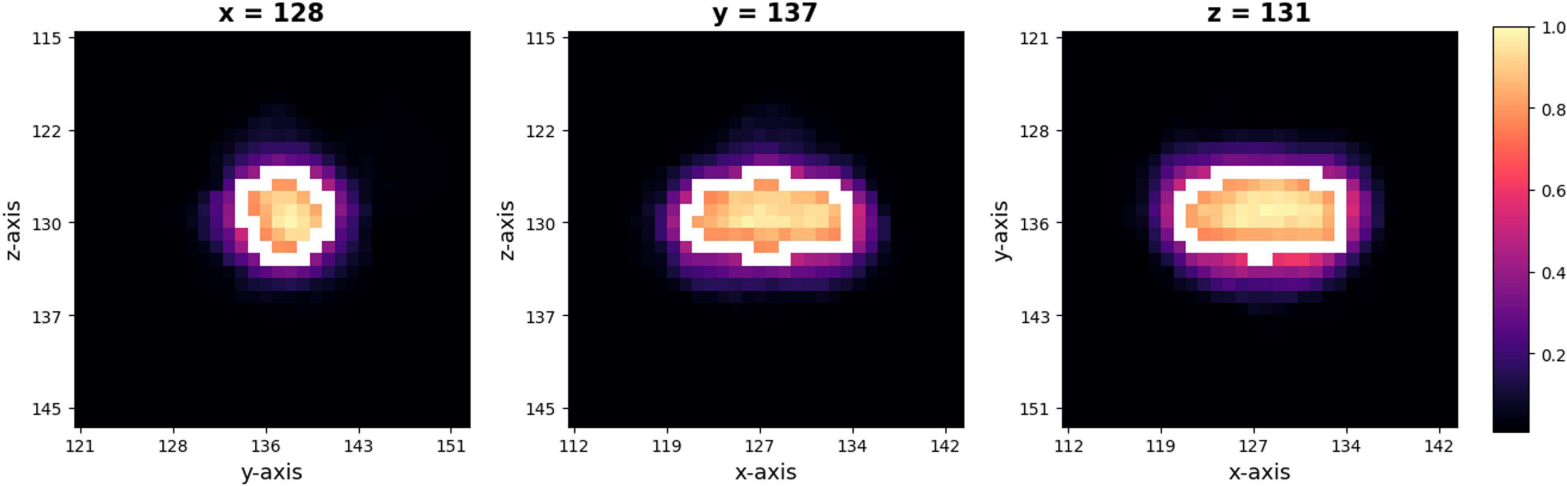
Selected patch with 32 × 32 × 32 size from predefined coordinates (x = 128, y = 137, z = 131). A probability map of pituitary gland segmentation. White line indicates the boundary of an averaged pituitary gland and the colormap reflects the probability map derived from all training datasets.

**Figure 4:**
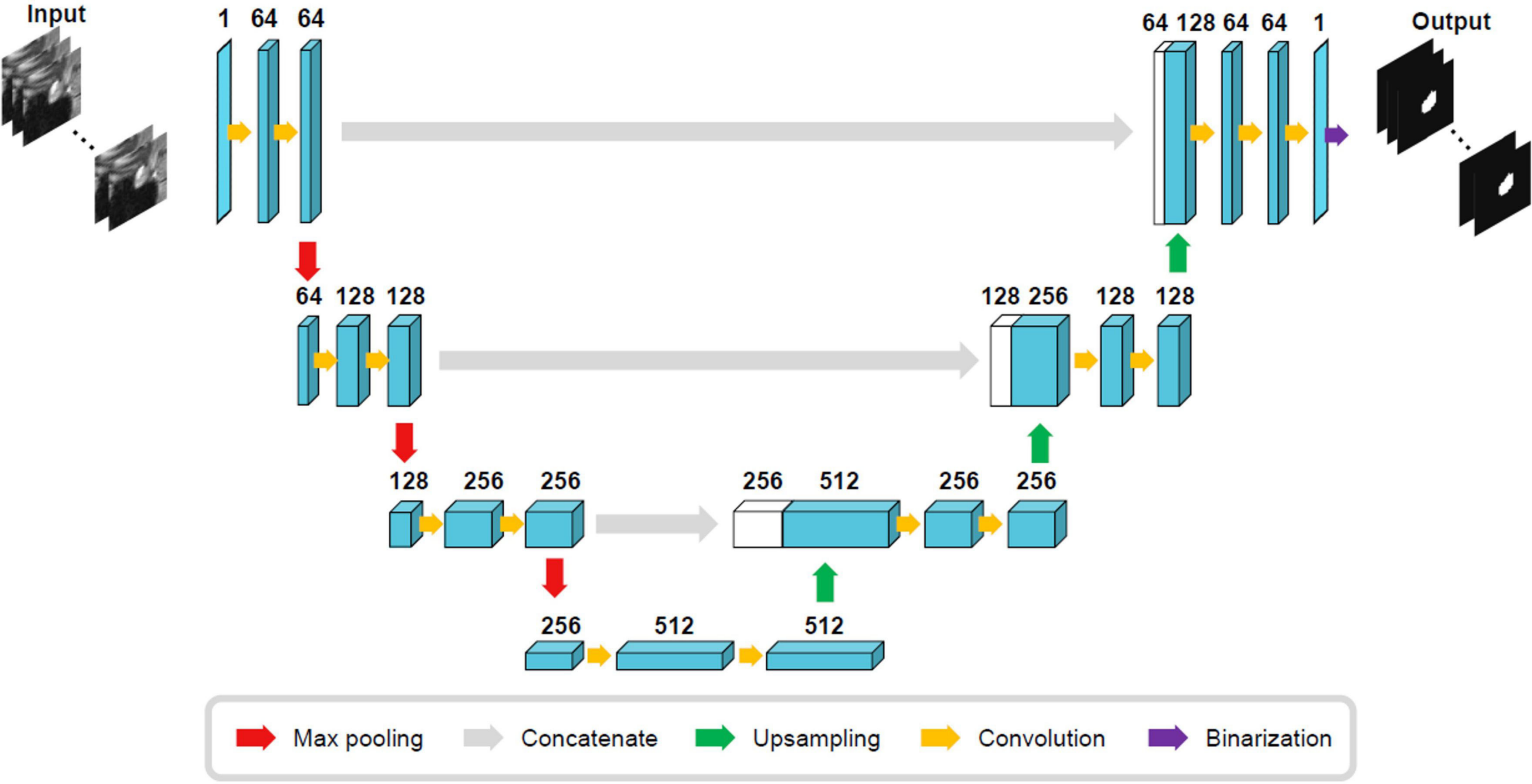
Three-dimensional U-Net architecture.

The size of the training ROI for the PG was constrained to a 32 × 32 × 32 voxel cube (**Figure 2**), centered around predefined coordinates. The predefined coordinates were estimated by averaging 143 MRI images of manually segmented PGs (**Figure 3**). To train our model, we utilized the 3D U-Net architecture (39), with a batch size of 16, for a total of 50 epochs, and an initial learning rate of 0.001 (**Figure 4**). The performance of the training model was evaluated through DICE loss, IoU, and DICE during the five-fold cross-validation.

### Model evaluation

Using an independent test dataset comprising of 10 participants, we assessed our model. We generated a confusion matrix and an average receiver operating characteristic curve (ROC) curve using the scikit-learn Python package. Furthermore, we calculated the average accuracy, precision, recall, and F1-score of the trained model.

### Regression analysis of PG morphologies with chronological age

We employed the proposed model to an external dataset consisting of 29 participants of varying chronological ages to examine the effect of age on PG morphometry. We computed two morphometric measures: the area of the mid-sagittal PG and the volume of the entire PG. One participant was excluded due to an abnormal brain size among 29 participants via interquartile range method (40). Subsequently, a linear regression analysis was performed, adjusted for sex, to assess the relationship between chronological ages and the PG morphologies. The statsmodels python package was used for the analysis (41).

## Results

**Figure 5:**
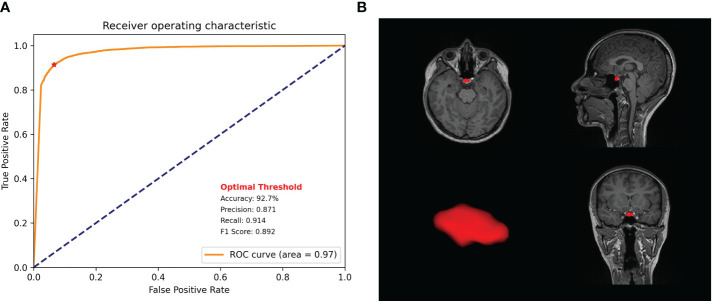
Model performance. **(A)** Receiver operating characteristic curve of the proposed model with precision, recall, f1 score, and accuracy. **(B)** Pituitary gland segmentation using the proposed model.

Our model showed a robustly high performance, as evidenced by the high average DICE and IoU scores and low DICE losses during the five-fold cross-validation (**Table 1**). The average DICE losses gradually declined from 0.14 in the first fold to 0.02 in the final fold. Concurrently, both the average IoU and DICE scores increased from the first fold to the final fold, reaching 0.95 and 0.98 respectively. The final model that demonstrated the lowest loss and highest DICE and IoU scores was evaluated using independent test datasets of 10 participants. The model revealed an accuracy of 92.7%, a precision of 0.87, a recall of 0.91, and an F1 score of 0.89, representing an ROC area of 0.97 (**Figure 5**).

**Table 1:** The averaged performance of the training model during the five-fold cross-validation.

Fold	Averaged Loss	Averaged IoU	Averaged DICE
1	0.14	0.72	0.83
2	0.07	0.85	0.92
3	0.04	0.90	0.95
4	0.03	0.93	0.96
5	0.02	0.95	0.98

**Figure 6:**
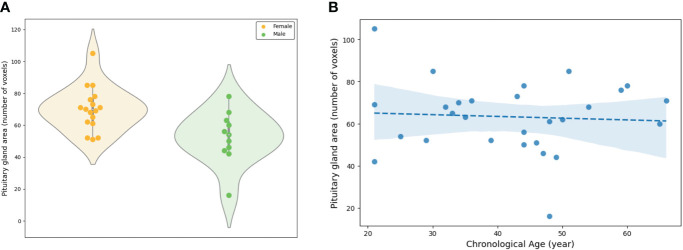
Evaluation of the area of the mid-sagittal pituitary gland involved: **(A)** A comparison between the female and male groups, and **(B)** an assessment of the association between the area and chronological ages.

**Figure 7:**
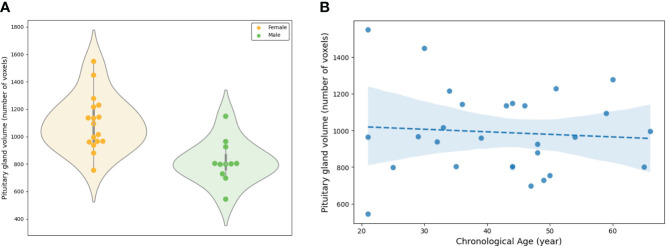
Evaluation of the volume of the entire pituitary gland involved: **(A)** A comparison between the female and male groups, and **(B)** an assessment of the association between the volume and chronological ages.

In PG morphometry analysis, significant differences were found between male and female groups in both the area of the mid-sagittal PG and the volume of the entire PG (p < 0.005 for the area and p < 0.001 for the volume) (**Figures 6A**, **7A**). The female group showed a significantly larger PG in terms of both area and volume compared to the male group.

The linear regression adjusted for sex showed statistical significance in both the area of the mid-sagittal PG and the volume of the entire PG (p = 0.018 for the area and p = 0.003 for the volume) (**Figures 6B**, **7B**). Both the area and volume revealed significant negative relationships with the chronological ages (coefficient = -0.02 for the area and coefficient = -0.46 for the volume).

## Discussion

Our model revealed robustly high performances in PG segmentation, as shown from the accuracy, precision, recall, and F1 scores. The high performance of the proposed model could be achieved via several strategies designed to overcome the limitations of the 3D U-Net architecture, which requires high-specification GPU memory. First, we constrained the training data size to 32 × 32 × 32 with a predefined location of the center voxel. The PG is a small brain structure of <10 mm (21) and located at the base of the brain near the optic chiasm with minimal variability in its location (**Figure 3**). Patching the training data can decrease the time for training the model to acquire the best performances and the GPU memory requirements, as suggested in a previous study (42). Secondly, data augmentation overcame the small sample size of the training model and enhanced the model performance. We adopted a random affine transformation and elastic deformation that have been widely used to train models (43). Our augmented data revealed a slightly different shape and size without unanticipated changes (**Figure 1**). It was expected that our data augmentation would allow us to better represent realistic data patterns leading to high segmentation performance for new data.

The proposed model accurately segmented the PG from new data, and the segmented PG morphology from the external dataset demonstrated a significant association with the chronological age. Two PG morphologies, one is an area of the mid-sagittal PG, and the other is the volume of the entire PG, were associated with sex and age, that is, both the area and volume exhibited significant differences between the female and male groups. This is supported by previous studies that reported that sex-specific morphometry was derived from different hormone systems (5, 44, 45). Furthermore, the negative association of those morphologies with chronological age was also consistent with previous reports (44, 46, 47) in which the height and cross-sectional area of the PG were greater in young adults than in old adults, which reflected the chronological changes of the endocrine activity. The area and volume demonstrated similar results although significant associations of the height in the PG may not be found due to the smaller sample size of the regression analysis than previous studies (44, 47).

This study has some limitations. First, we did not compare the performances of the segmentation model among various deep-learning architectures such as the fully convolutional network or VNet (48). The proposed model could be improved by adopting other deep-learning strategies with optimal hyperparameters although the current model revealed excellent performance. Secondly, our model was trained by a limited participant group of normal adults. The proposed model’s robustness in segmenting a wide range of new data types can be enhanced through further training with new datasets, such as those from the elderly and children. The inclusion of various MRI images of the PG with different resolutions, contrasts, and orientations may contribute to the validity of the segmentation in terms of qualitative and quantitative aspects.

Overall, despite the study limitations, our novel model demonstrates significant potential for the estimation of health conditions (49). The evaluation of a health condition is crucial for patients to maintain or improve their quality of life through early prevention and intervention. For example, individuals concerned about obesity could undergo an MRI scan to assess any abnormalities in the volume or height of the PG. Patients exhibiting greater abnormalities compared to controls could undergo interventions to prevent worsening health conditions. Furthermore, the capability of our current model, which has been trained using data from normal adults, can robustly and efficiently segment a large volume of PG data, which could potentially be utilized to construct a normal distribution of PGs (21). This type of distribution can render the identification of a biomarker for obesity, which is one of the most significant societal challenges (5), and a biomarker for the growth hormone system for child development that makes detection of hormonal changes possible through the evaluation of the PG morphometry.

In summary, our novel method makes automatic segmentation of the PG possible while demonstrating high performance and significant potential for precise volumetric analysis of the PG. The proposed model can be used to derive a crucial biomarker from anatomical MRI data for various endocrine-related health conditions, which may lead to the development of an accurate assessment tool to enhance the quality of life by facilitating early detection and intervention for endocrine disorders.

## Data availability statement

The raw data supporting the conclusions of this article will be made available by the authors, without undue reservation.

## Ethics statement

The study was approved by the Institutional Review Board of Tohoku Fukushi University. The studies were conducted in accordance with the local legislation and institutional requirements. The participants provided their written informed consent to participate in this study.

## Author contributions

U-SC: Conceptualization, Data curation, Formal Analysis, Investigation, Methodology, Software, Validation, Visualization, Writing – original draft, Writing – review & editing. Y-WS: Conceptualization, Data curation, Funding acquisition, Investigation, Project administration, Resources, Supervision, Writing – original draft, Writing – review & editing. SO: Funding acquisition, Investigation, Project administration, Resources, Supervision, Writing – review & editing.
